# Lung Inflammatory Genes in Cystic Fibrosis and Their Relevance to Cystic Fibrosis Transmembrane Conductance Regulator Modulator Therapies

**DOI:** 10.3390/genes14101966

**Published:** 2023-10-20

**Authors:** Annalucia Carbone, Pamela Vitullo, Sante Di Gioia, Massimo Conese

**Affiliations:** 1 Department of Clinical and Experimental Medicine, University of Foggia, 71122 Foggia, Italy; annalucia.carbone@unifg.it (A.C.); sante.digioia@unifg.it (S.D.G.); 2 Cystic Fibrosis Support Center, Ospedale “G. Tatarella”, 71042 Cerignola, Italy; pamelavitullo@gmail.com

**Keywords:** cystic fibrosis, gene polymorphisms, CFTR, CFTR modulator therapy, lung disease, airway epithelial cells, macrophages, neutrophils

## Abstract

Cystic fibrosis (CF) is a monogenic syndrome determined by over 2000 mutations in the *CF Transmembrane Conductance Regulator* (*CFTR*) gene harbored on chromosome 7. In people with CF (PWCF), lung disease is the major determinant of morbidity and mortality and is characterized by a clinical phenotype which differs in the presence of equal mutational assets, indicating that genetic and environmental modifiers play an important role in this variability. Airway inflammation determines the pathophysiology of CF lung disease (CFLD) both at its onset and progression. In this narrative review, we aim to depict the inflammatory process in CF lung, with a particular emphasis on those genetic polymorphisms that could modify the clinical outcome of the respiratory disease in PWCF. The natural history of CF has been changed since the introduction of CFTR modulator therapies in the clinical arena. However, also in this case, there is a patient-to-patient variable response. We provide an overview on inflammatory/immunity gene variants that affect CFLD severity and an appraisal of the effects of CFTR modulator therapies on the inflammatory process in lung disease and how this knowledge may advance the optimization of the management of PWCF.

## 1. Introduction

Although a syndrome with clinical effects on many organs, cystic fibrosis (CF) morbidity and mortality are determined by lung disease [1,2,3]. CF lung disease (CFLD) is characterized by opportunistic microorganism colonization and infection as well as by chronic inflammation, which involves epithelial cells, innate immune cells (macrophages and neutrophils), and adaptive immune cells (various types of T cells) [4,5,6].

CF is due to one single gene, *CFTR* (*CF Transmembrane Conductance Regulator*), codifying a channel implied in chloride and bicarbonate secretion at the level of epithelial surfaces. The *CFTR* gene can harbor more than 2000 mutations [7], which can be classified in six classes, depending on the lack or dysfunction of the CFTR protein [8] (Table 1). Briefly, class I includes premature termination codons, which hamper the synthesis of a functional full-length protein or determine the decay of CFTR mRNA. Class II mutations comprise those altering the correct trafficking of the protein to the plasma membrane and include the most frequent one, *F508del*. In class III, we find mutations altering the capacity of channel activation (gating defects). Class IV mutations decrease channel conductance. Mutations comprised in class V are those determining a reduction of mRNA levels. Class VI mutations cause the reduction of the half-life of CFTR when trafficked onto the plasma membrane.

At the early stages of CFLD, there is a reduction in chloride secretion in the airway surface fluid, which, together with the accompanying sodium and fluid hyperabsorption due to epithelial sodium channel (ENaC) hyperactivation, is responsible for a sticky viscous mucus and a dramatic decrement in the mucociliary clearance [9], which in turn is responsible for the colonization and subsequent infection by opportunistic pathogens. *Haemophilus influenzae* and *Staphylococcus aureus* appear early during infancy and early childhood, whereas Gram-negative bacteria, including *Pseudomonas aeruginosa* and the *Burkholderia cepacia* complex, take over later on, although the CFTR modulator therapy is changing this epidemiology [10]. The impairment of bicarbonate secretion by the lack of a functional epithelial CFTR contributes to these events due to the decrease in airway surface fluid pH and, hence, in the breach of two important barriers to infections, i.e., the mucociliary clearance and the activity of antimicrobial peptides [11,12,13,14,15]. In subsequent steps, CFLD is derived from alterations in inflammatory responses that drive the damage of airway structures, namely, collagen and elastin, leading to bronchiectasis and producing a vicious cycle between inflammation and infection [16]. In this destructive process, neutrophil- and macrophages-derived mediators, essentially serine proteases (e.g., elastase), cysteine proteases (e.g., cathepsins), and metalloproteases (e.g., MMP-9), contribute to the degradation of airway structures and their ensuing remodeling [17,18,19].

### 1.1. Immunoinflammation in CFLD

CFLD is basically a mucosal immunodeficiency disease whose hallmarks are epithelial innate immune dysfunction, oxidative stress, alterations in resident and recruited immune cells, and remodeling of the airways [20,21]. Thus, the involvement of the immune response, including inflammation, is crucial in the CFLD pathogenesis in regard to innate immunity and barrier function branches. Soluble mediators, such as the collectins mannose binding lectin (MBL) and surfactant proteins (SPs), play the role of ante-antibodies, neutralizing airway pathogens and microbial products, thus allowing for their disposal by the complement system [22,23]. The insufficiency of collectins (MBL and SP-D) is associated with the early acquisition of the *P. aeruginosa* infection [24]. Airway epithelial cells are dysregulated in CF, being responsible for the secretion of pro-inflammatory cytokines as well as chemokines recruiting innate-immune cells (e.g., polymorphonuclear neutrophils [PMNs]) [25,26,27]. PMNs are found accumulated within the airways where they are disabled in their capacity to remove infective agents; nevertheless, their activation is a hallmark of CFLD, together with increased concentrations of pro-inflammatory mediators, including tumor necrosis factor-α (TNF-α), interleukin (IL)-1β, IL-6, IL-8, IL-17, IL-33, and granulocyte-macrophage colony-stimulating factor (GM-CSF) [28].

Innate immunity also appears deregulated concerning both macrophages and PMN subclasses being increasingly recognized [29,30]. CF macrophages entail defects in the intracellular killing of phagocytosed microorganisms and a heightened cytokine production, which is likely related to a lack of CFTR or CFTR dysfunction, contributing to the alteration of bacterial removal from the airways and inflammatory CF milieu [31,32,33,34]. Moreover, CF monocytes have been found to constitutively secrete MMP-9 [35]. In the case of CFTR involvement in phagolysosome acidification and ensuing intracellular killing of pathogens by phagocytes, it is worth mentioning that this is a controversial issue, since opposite results were obtained, i.e., a non-CFTR pathway to acidification or even a lack of acidification in CF monocytes/macrophages [36,37,38], demonstrating how different models and investigation techniques may compound the already intrinsic patient-to-patient variability.

On the other hand, the analysis in CF secretions reveals functionally different subsets of PMNs, including populations with abnormal immune function and defective bacterial killing [29]. Moreover, PMNs are metabolically reprogrammed when recruited and activated in CF airways [39]. A study on CF sputum, based on single-cell RNAseq, revealed that immune cells are spread along a spectrum of functional conditions, leading to the identification of different mononuclear phagocyte populations whose gene signature is consistent with excessive inflammation and impaired host defense responses occurring in CF airways, and PMN archetypes with an immature or proinflammatory phenotype not being capable of properly recognizing and removing pathogens [40]. Heterogeneity in immune cell transcriptomic profiles may underlie subjects-specific differences in CFLD progression and response to therapy.

Cellular receptors are used to sense either pathogen-associated molecular patterns (PAMPs) or danger-associated molecular patterns (DAMPs) in the airways. Toll-like receptors (TLR) are pattern recognition receptors (PRR) that ligate and discriminate an array of microbial antigens (see below for a list, Section 2.2), so their dysregulation has been implicated in the lung inflammation of PWCF [41]. The receptors for advanced glycation end products (RAGEs) are expressed on macrophages, endothelium, and lymphocytes; ligate the S100 family of calcium-binding proteins as well as the high-mobility group protein B1 (HMGB-1); and activate intracellular signaling pathways for the dependent secretion of different cytokines [42,43,44,45].

After the engagement of cellular receptors, two main transduction signaling pathways are at work in innate-immune cells, including airway epithelial cells, at the onset of CFLD, namely, the nuclear factor kappa-light-chain-enhancer of activated B cells (NF-κB) and inflammasome pathways. The NF-κB signaling can be stimulated either by the CFTR-mediated dysfunction of ionic cytosolic milieu or by DAMPS/PAMPs, driving the production of many inflammatory mediators, including cytokines (such as, for example, TNF-α, IL-6, and IL-8) [46]. On the other hand, it is the Na^+^ and K^+^ imbalance inside airway epithelial cells and monocytes that, together with reactive oxygen species (ROS) and endoplasmic reticulum (ER) stress, activates the nucleotide-binding domain, leucin-rich repeat family pyrin domain containing 3 (NLRP3) inflammasome, which leads to excessive production of IL-1β and IL-18 [46,47,48,49]. Airway epithelial cells and monocytes bearing different CF-associated mutations displayed increased NLRP3-dependent IL-1β/IL-18 secretion, an effect reversed by either NLRP3 inflammasome pathway inhibitors or small-molecule inhibitors of ENaC [47]. The increase in IL-1β in the bronchoalveolar lavage fluid (BALF) of PWCF may be due to the activation of neutrophil NLRP3 inflammasome [39].

In regard to all these immune responses, the CFTR role in the onset of the CF inflammatory alterations is still quite controversial. Some recent studies highlighting the function of the CFTR protein in immune cells have proposed CF as an autoinflammatory disease based on the aberrant activation of the innate immune system, further enhanced by the harsh mucosal environment already burdened by the chronic colonization of opportunistic pathogens [47,50]. Thus, there will be an increased production of pro-inflammatory cytokines (such as TNF-α, IL-1β, IL-6, IL-17, and IL-18), which would activate and perpetuate immune cells’ activation. Interestingly, in young children with CF, inflammation (IL-1β) can be detected in BALF in the absence of infection [51], a finding that confirms previous observations in patients and animal models with CF [52,53,54]. Whatever the mechanistic role of CFTR or other determinants in the onset of the innate immune response in CFLD, the ensuing chronic inflammation is responsible for the progression of lung damage apparently more than infection [55].

A few studies have interrogated the dysfunction of adaptive immunity in the context of CFLD [4,56,57,58]. It can be assumed that innate immune cell alterations precede those of B and T cells; thus, for the aim of this review, we will focus on innate-immune cell and mediator dysfunction and dysregulation. However, it is worth mentioning that adaptive immunity contributes to dysregulated innate immunity. Indeed, upon sensing bacterial pathogens, DCs induce the differentiation of T lymphocytes into Th17 cells, which produce IL-17A and IL-17F, indirectly increasing neutrophil recruitment and activation in the airways by regulating cytokines that mediate granulopoiesis (GM-CSF) as well as the local production of CXCR2 ligands that are chemoattractants for neutrophils [59,60]. IL-17A, IL-17F, and IL-23 (an ancillary cytokine serving Th17 cell differentiation) have high levels in the plasma and airway secretions of PWCF [61,62].

### 1.2. CFTR Modulators: A New Paradigm in CF Treatment

A paradigm shift in CF treatment has emerged since 2012. In this year, the first CFTR modulator, i.e., ivacaftor (VX-770 or Kalydeco) was approved by the FDA to treat patients with class III mutations (e.g., *G551D*) [63]. The CFTR modulators are those small-molecule drugs capable of modifying the pathological behavior of mutated CFTR proteins [64,65,66] (Table 1). Ivacaftor is a so-called potentiator, which allows for the increase in the CFTR channel’s open probability when the protein is present at the cell membrane. Besides class III mutations, ivacaftor is also effective in class IV variants [67]. Correctors are such drugs inducing the increment in bulk CFTR protein on the plasma membrane, avoiding the entrapment of class II mutated CFTR in the ER and its premature degradation. Thus, ivacaftor was administered together with correctors (lumacaftor [VX-809] first and then tezacaftor [VX-661]), and this combination resulted in clinical efficacy in homozygous *F508del* patients, heterozygous for *F508del* and *G551D*, or heterozygous for *F508del* and a minimal function mutation [68,69,70,71]. A further advancement was the triple therapy comprising ivacafor, tezacaftor, and elexacaftor (VX-445), which was approved in PWCF who had a single *F508del* allele, i.e., 90%, and which is also recalled as a highly effective modulator therapy (HEMT) [72,73]. For class V and class VI mutations, there is no agent approved for the clinical use, although mRNA amplifiers (which increase the amount of mutant CFTR messenger RNAs) and stabilizers (which decrease surface protein turnover) are in development, respectively [64]. Finally, class I mutations are also in this sense orphan ones, with read-through agents (which suppress premature stop codons), nonsense-mediated decay pathway inhibitors, and gene therapy approaches in preclinical research [74].

There is a great variability in CFLD progression along CF patients with the same mutational asset. The cause of this heterogeneity has been imputed to the presence of genetic and environmental modifiers [75,76]. Moreover, the clinical response to small molecules targeting the CFTR abnormalities is subjected to a kind of heterogeneity [77,78,79] and is not sufficient to completely normalize the physiology of affected organs, including the lung [80]. The study of gene variants by different approaches may shed light on the inflammatory/immune process in the CF airways as well as the heterogeneous CFLD progression and response to CFTR modulator therapies.

## 2. Immune Gene Variants Involved in CFLD Pathogenesis and Progression

To elucidate the variable severity of clinical manifestations of CF, many studies have focused their attention on the discovery of genetic variants or polymorphisms located outside the *CFTR* locus and called “modifier genes” altering the severity of CFLD [75,81,82,83]. A list of these genes, which belong to inflammation/immunity, include: *MBL*, *NOS* (nitric oxide synthase), *SERPINA1* (α1-antitrypsin), *HLA* (human leukocyte antigen), *TNFA*, *GST* (glutathione S-transferase), *TGFB1*, *ACE* (angiotensin 1 converting enzyme), *IL10*, *MIF* (Macrophage Migratory Inhibitory Factor), and *FcγRIIA* (Fc Receptor IIA). To search for modifier genes in CF, two approaches have been applied: a candidate-gene approach based on the pathophysiology of the phenotype; and an approach performed using whole-genome analysis, particularly GWAS for a genome-wide association study or WGS for whole-genome sequencing, and WES for whole-exome sequencing [81]. In CFLD, lung function, usually estimated through measurements of the forced expiratory volume in one second (FEV_1_), is the most studied phenotype in the search for modifier genes; in this sense, the candidate-gene approach identifies modifier genes based on their physiological role in lung function: genes involved in tissue repair, host defense and inflammation, epithelial surface ion transport and mucus secretion, and response to drug therapy. Regarding the whole-genome analysis-based approach, GWAS were conducted, which identified seven genomic regions of interest associated with the severity of lung function [75]. We focused on variants of inflammatory/immune genes altering CF lung disease severity and described in the last 15 years (Table 2).

### 2.1. Collectins

A pulmonary surfactant is compounded by lipids and proteins that are considered a kind of “coating” that covers the alveolar surface. By reducing surface tension at the air–liquid interface, this bioactive complex prevents alveoli from collapsing after expiration. The protein component is constituted by four specific proteins, which have been described and are named surfactant protein A (SP-A), SP-B, SP-C, and SP-D. These proteins have been shown to play essential roles in the regulation of surfactant lipid metabolism, in the organization of lipid membrane, and in the host defense of the lung. SP-B and SP-C are extremely hydrophobic and play crucial roles in biophysical functions of surfactants. SP-A and SP-D are water-soluble and are members of the collectin subgroup of the lectin superfamily, together with MBL. Collectins are well acknowledged to be key members that constitute innate immunity. Pulmonary collectins such as SP-D and MBL have been identified as principal factors in the innate host immunity and modulation of immune response to pathogens in the lung [84]. The specific interactions of lung collectins with microorganisms lead to opsonization, complement activation (MBL), growth inhibition, and viral neutralization. Moreover, lung collectins bond directly with macrophages and stimulate the phagocytosis or clearance of colonizing microorganisms highly dependent on ROS. In CF, lung function and innate immunity alterations are strictly correlated with genetic variants of SPs and MBL levels [85,86].

An interesting study investigated *MBL2* genotypes and diplotypes in a cohort of 1019 CF pediatric patients homozygous for *F508del* [87]. While a haplotype is a combination of alleles at multiple loci that are transmitted together on the same chromosome, a diplotype is a matched pair of haplotypes on homologous chromosomes [88]. The “low *MBL2*” diplotype group (Y0/Y0, X0/Y0, and XA/Y0; where Y is the wild-type variant -221G in the promoter, X is the variant -221C at the same location, and A comprises the wild-type variants 154C, 161G, and 170G at Exon 1) was associated with a significantly earlier onset of *P. aeruginosa* infection and a steeper decline in FEV_1_. Interestingly, these effects were magnified in the presence of high-expressing *TGFB1* variants.

Lin et al. [89] investigated the genetic contribution of *SFTPA1*, *SFTPA2*, *SFTPB*, *SFTPC*, and *SFTPD* genes to CF and lung disease severity; they identified, between single-nucleotide polymorphisms (SNPs) and several intragenic and intergenic SNP-SNP interactions, a single *SFTPB* SNP that was associated with a mild CF (rs7316), and several intergenic interactions that are associated with either mild or moderate/severe CF were observed (Table 2). Therefore, the authors demonstrated that complex SNP-SNP interactions of the SP genes may conduce the pulmonary disease in CF patients and speculated that *SFTP* SNPs may contribute as modifiers for the different progressions of pulmonary disease in CF and/or may be the basis of its severity.

### 2.2. Cellular Receptors

Innate immunity depends on a series of germline-encoded, invariant receptors called PRRs. These receptors, including Toll-like receptors (TLRs), are devoted to detecting infectious organisms and to prime an acute inflammatory response. TLRs are a class of proteins that can acknowledge and distinguish different types of microbial antigens such as: proteins (e.g., flagellin from bacterial flagella), lipoteichoic acid (LTA) and peptidoglycan (PGN) from Gram-positive bacteria, lipopolysaccharide (LPS) from Gram-negative bacteria, lipoarabinomannan (LAM), lipopeptides, lipoglycans, and lipomannans from mycobacteria, zymosan from yeast, double-stranded (ds) RNA of viruses, and DNA from viruses and bacteria. Following their activation by these factors, TLRs transduce intracellular signals to control pro-inflammatory gene expression. These receptors signal via several kinases and adaptor proteins MyD88/Mal or TIR domain-containing adaptor inducing IFN-β (TRIF)/TIR domain-containing adaptor molecule-1, IL-1R-associated kinases, TNFR-associated factor 6, TGF-β-activated kinase 1, and IκB kinases to activate NF-κB and induce the expression of NF-κB-regulated genes [90]. The innate immune system is pivotal to the inflammatory response occurring in the lung in the context of CF. Haerynk and colleagues [91] studied the effect of polymorphisms of TLRs on the severity of CF lung disease. They demonstrated that mutant alleles of SNPs *TLR2* rs1898830 and rs5743708 are associated with the rapid diminution of FEV_1_. Polymorphism *TLR2* rs1898830 may be evidence of the most important association with lung function decline in contrast to the other two SNPs of *TLR2* rs3804100 and rs5743708, both with a low frequency of homozygote mutant genotypes. TLR-2 in cooperation with TRL-1 as a heterodimer plays an important role to discriminate and to mediate signaling in response to different microbial lipoproteins and lipopeptides. Therefore, *TLR2* as well as *TLR1* polymorphisms are linked to FEV_1_ decline. Haerynk et al. [91] also demonstrated that patients being heterozygous for *TLR1* polymorphism rs5743551 in the promoter were more probably associated with mild lung disease when compared to patients being homozygous for the wild type A allele. Furthermore, CF patients homozygous for the variant C allele of *TLR5* polymorphism (exon 6) were more often associated with a strong decline in %FEV_1_ compared to heterozygous patients (Table 2). In this study, they used FEV_1_ decline as an outcome parameter, which is more informative than mean FEV_1_ [91].

Regarding TLR-5, Blohmke et al. [92] studied the association among *TLR5* polymorphism, rs5744168 (c1174 C > T), and FEV_1_ decline; they could not demonstrate a significant modifying effect due to this polymorphism on the mean FEV_1_ in adult and pediatric CF patients.

Beucher and colleagues [93] examined their hypothesis whether *AGER*, the gene encoding the receptor for advanced glycation end products (RAGEs), was associated with a decline in lung function in a cohort of PWCF from the French CF Gene Modifier Study. The promoter *AGER*-429T/C variant was associated with poorer lung function as compared to the patients homozygous for the major allele (*AGER*-429TT).

### 2.3. Inflammasome

Inflammasomes are multimeric protein complexes that act as a shelf for caspase activation and pro-inflammatory cytokine release. Activated inflammasomes cause pro-inflammatory cytokine release and Gasdermin D-mediated pyroptotic cell death, allowing for the activation of fundamental defense mechanisms, such as cell migration [94]. NLRP3 is one of the inflammasomes expressed in dendritic cells, monocytes, macrophages, neutrophils, and epithelial cells. NLRP3 inflammasome activation needs the combination of two signals: priming and activation. The priming signal is triggered by PRRs, such as TLR-4, and leads to the NF-κB-mediated transcription of NLRP3, pro-IL-1β, and pro-IL-18. The same signal also results in the licensing of NLRP3, which involves the regulation of post-translational modifications, protein–protein interactions, and cellular localization of NLRP3, and which can be used to modulate its activity. The activation (second) signal can be provided by a variety of events (stimuli including extracellular ATP, pore-forming toxins, RNA viruses, and particulate matter; multiple molecular or cellular events, including ionic flux, mitochondrial dysfunction and ROS generation, and lysosomal damage) that lead to the disruption of cellular homeostasis and stimulate the aggregation of the inflammasome complex. At the moment of the activation, NLRP3 undergoes oligomerization, an event fundamental for interaction with the apoptosis-associated speck-like protein containing a caspase-recruitment domain (ASC) adapter protein and recruitment of pro-caspase-1. This recruitment leads to the cleavage and activation of caspase-1, which in turn cleaves the cytokine precursors pro-IL-1β and pro-IL-18 and produces active cytokines. Ultimately, the activation of the NLRP3 inflammasome implies the release of these pro-inflammatory cytokines [94].

In recent years, the NLRP3 inflammasome has also been implicated in inflammation that characterizes cystic fibrosis. The production of IL-1β, an active cytokine, from its precursor, pro-IL-1β, is mediated by NLRP3 inflammasome expressed by neutrophils. In addition, it is responsible for the cytokine explosive release in the lungs during the pathogenesis of CF [95].

The NLRC4 inflammasome is activated by the flagellin of Gram-positive and Gram-negative bacteria and by the type III secretion system (T3SS) of Gram-negative bacteria. NLRC4 produces, through the NF-κB pathway, IL-1R antagonist (IL-1Ra), which binds IL-1β, delaying the progression of fibrogenesis. The cooperation between NLRP3 and NLRC4 inflammasomes has been also reported in a CF mouse model in alveolar macrophages and neutrophils [49]. Graustein et al. [96] examined candidate genetic variants in the NLRP3 and NLRC4 inflammasome pathway to study the correlation between chronic *P. aeruginosa* infection and lung function in a large cohort of children with CF. They found two variants, one in *NLRP3* (p.Q705K) and one in *NLRC4* (p.A929S), with significant associations with clinical parameters in this cohort (Table 2). They then studied the mechanistic impact of these variants in human macrophage-like cells to test their hypothesis that hyper-inflammatory inflammasome variants are harmful in CF and that hypo-inflammatory variants are protective [96].

### 2.4. Cytokines

CF lung disease is characterized by an inflammatory state, which has been described in vitro in cell systems, in vivo in a murine model, and ex vivo in samples from CF patients. As a consequence, many studied candidate genes were derived from the field of immunity, immunology, and host defense such as the cytokines *IL8*, *IL1B*, *IL10*, *TNFA*, and *TGFB1* [97]. IL-8, the major chemokine-recruiting neutrophils during inflammation, is responsible for the excessive infiltration of neutrophil in CF airways. It was clearly demonstrated [98,99] that polymorphisms within *IL8* were associated with CF pulmonary disease severity: in particular, in CF groups analyzed by Hillian et al. [98], the rs4073A allele is protective and the rs4073T allele is associated with the more acute phenotype. Regarding *IL1B*, several polymorphisms have been found: rs2227306 and rs2227307 [100], rs3917356 and rs4848306 [101], and rs16944 [99]. All these polymorphisms were associated with severe lung diseases (Table 2). de Vries et al. [99] also analyzed the *IL10* polymorphism rs1800896, and they were able to demonstrate significant evidence for a correlation between *IL10* genotype and a more severe lung disease. The gene products TNF-α and TNF-β, also known as lymphotoxin-α (LT-α), exhibit a large spectrum of inflammatory and immunomodulatory activities. In particular, TNF-α stimulates phagocytosis and the production of PGE2 by macrophages. Furthermore, TNF-α is a potent chemoattractant, supporting neutrophils during migration across endothelial cells. The effects of LT-α are similar, essentially to TNF-α, but LT-α is also crucial for the development of lymphoid organs. It is clear that both cytokines play a pivotal role in the pathogenesis of many inflammatory diseases, including cystic fibrosis [102]. Shmarina and colleagues investigated the possible roles of *TNFA* gene polymorphisms in CF disease phenotype and progression. They could not find any association between *LTA* or *TNFA* single SNPs and lung function in a CF cohort, despite finding that neutrophil elastase activity was higher in sputum samples from patients bearing the polymorphisms *TNFA*–308GA or *LTA* +252GG. In the same cohort, carriers of both polymorphisms did have an improved lung function as opposed to subjects from other *TNFA*—*LTA* genotype groups [103].

In the context of inflammatory processes, specifically during the chronic pulmonary disease, TGF-β1 produced by a bronchial epithelial cell, acts by regulating the recruitment and activation of neutrophilic granulocytes within a complex network of inflammatory and anti-inflammatory cytokines. In a recent study, Sagwal et al. showed that the levels of serum TGF-β1 were increased in pulmonary exacerbation phases, infection with *P. aeruginosa*, and in subjects with at least one *F508del* mutation [104]. To support these data, they demonstrated that TGF-β1 levels decreased significantly after an antibiotic treatment of pulmonary exacerbations [104]. Notably, this cytokine has been identified as a genetic modifier for CF lung pathology. By using spirometry as a marker of pulmonary function in a large cohort of pediatric CF patients, Corvol et al. [105] were able to confirm previous data on adults with CF reporting a correlation between *TGFB1* and lung disease progression; in particular, variants of the *TGFB1* gene at position +869T/C resulted in a significant association with lung function reduction (Table 2). Finally, in Trojan’s work [106], three polymorphisms, rs1800469 located in the promotor region and rs1800470 and rs1800471 located in Exon 1 of the *TGFB1* gene, were analyzed. They established that *TGFB1* SNP genotypes, as modifiers of CF lung disease, can be associated with a faster decline in pulmonary function.

**Table 2:** Variants of inflammatory and immunity genes associated with CFLD.

Gene	Variant	Associated with	Reference
Soluble Mediators			
*MBL2*	Y0/Y0, X0/Y0, and XA/Y0 diplotypes	MBL2 deficiency, which was associated with more rapid decline of pulmonary function and enhanced by high-producing *TGFB1* gene variants	Dorfman et al., 2008 [87]
*SFTPA1*, *SFTA2*, *SFTB*, *SFTPC*, *SFTPD*	*SFTPB* SNP (rs7316)	Mild lung disease	Lin et al., 2018 [89]
	Intergenic interactions between *SFTPB* and *SFTPD*/*SFTA1*+*SFTPA2*	Mild lung disease	
	Intergenic interactions between *SFTPB* and *SFTPD*/*SFTA1*+*SFTPA2*	Moderate/severe lung disease	
	Intergenic interactions between *SFTPA1* and *SFTPA2*		
Cellular Receptors			
*TLR5*	c.1174C>T (rs5744168)	Improvements in lung function associated with the T allele were not statistically significant	Blohmke et al., 2010 [92]
*TLR5*	rs5744174	Extreme fast FEV_1_ decline	Haerynck et al., 2013 [91]
*TLR1*	Homozygous for rs5743551	Faster decline of FEV_1_ compared to heterozygous genotype	Haerynck et al., 2013 [91]
*TLR2*	rs1898830, rs5743708, and rs3804100	Lung disease severity	Haerynck et al., 2013 [91]
*AGER*	Variant −429T/C	More severe CFLD	Beucher et al., 2012 [93]
Inflammasome			
*NLRP3*	p.(Q705K)	A higher rate of *P. aeruginosa* colonization and worsened lung function	Graustein et al., 2021 [96]
*NLRC4*	p.(A929S)	A lower rate of *P. aeruginosa* colonization and protection from lung function decline	
Cytokines			
*IL8*	rs4073	Protective	Hillian et al., 2008 [98]
	rs2227306 and rs2227307	Severe lung disease	
*IL8*	rs4073, rs2227306, and rs2227307	Markers of severe lung disease	Furlan et al., 2016 [107]
*IL8*	rs4073	More severe lung disease	de Vries et al., 2014 [99]
*IL1B*		Severe lung disease	Stanke et al., 2011 [100]
*IL1B*	rs3917356 and rs4848306	Severe lung disease	Labenski et al., 2011 [101]
*IL1B*	rs16944	More severe lung disease	de Vries et al., 2014 [99]
*TNFA*	genotype TNF-α–308GA	Higher neutrophil elastase activity in sputum	Shmarina et al., 2013 [103]
*TNFA*	−857C/T polymorphism	Severe pulmonary phenotype	Hassanzad et al. [108]
*TNFR2*	+587T/G polymorphism	Severe pulmonary phenotype	Hassanzad et al. [108]
*IL10*	rs1800896	More severe lung disease	de Vries et al., 2014 [99]
*LTA*	+252GG polymorphism	Higher neutrophil elastase activity in sputum	Shmarina et al., 2013 [103]
*TGFB1*	+869CT	Less-pronounced rate of decline in FEV_1_	Corvol et al., 2008 [105]
	Homozygous TT genotype	High levels of sputum TNF-α	Trojan et al., 2022 [106]

CFLD: cystic fibrosis lung disease; FEV_1_: forced expiratory volume in one second.

## 3. Effects of CFTR Modulator Therapies on Inflammation/Immunity

**Figure 1:**
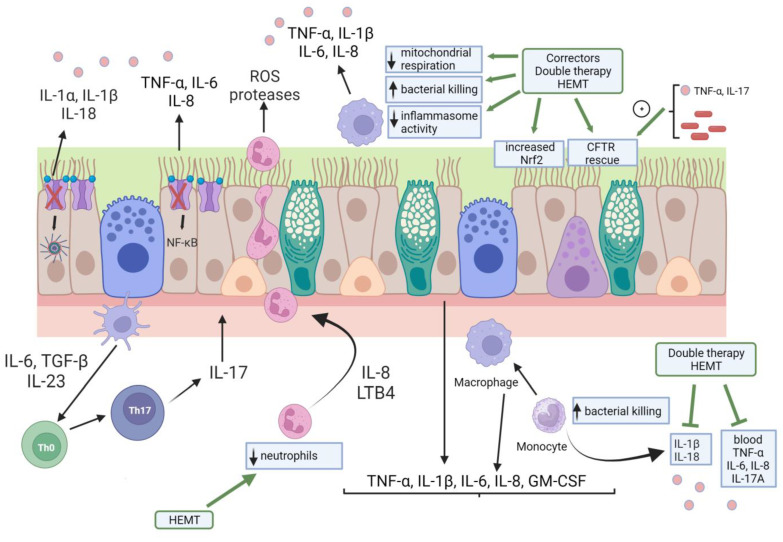
Pictorialization of CFLD pathogenesis and the effect of CFTR modulator therapy. Cellular landscape of CFLD pathogenesis include airway epithelial cells, monocytes/macrophages, and neutrophils. It should be noted that all these cytotypes express a functional CFTR, although it is only represented in airway epithelial cells here. Lack/dysfunction of CFTR in this last cell type leads to increased inflammasome and NF-κB activities with ensuing heightened cytokine production and release. These cytokines and chemokines stimulate neutrophil influx in the airways, a process amplified by the IL-23/IL-17 axis exerted by Th17 cells. Monocytes and macrophages are also hyper-activated in the CF airways, resulting in elevated secretion of cytokines in the blood and airway secretions. CFTR modulator therapies have resulted not only in the CFTR rescue but also in Nerf2 activation in airway epithelial cells. Inflammatory cytokines (TNF-α, IL-17) and bacterial material were shown to enhance CFTR rescue in airway epithelial cells by small-molecules drugs. Although with a certain variability among different dual therapies (ivacaftor/lumacaftor and ivacaftor/tezacaftor) and HEMT, monocytes and macrophages phenotype and functions were modified by CFTR modulators. Finally, HEMT was capable of diminishing neutrophils counts to normality. HEMT: highly effective modulator therapy.

Several steps in the pathogenesis, onset, and progression of CFLD are still unclear and controversial. The introduction of the CFTR modulator therapy may not only explain the pleiotropic effects of these drugs but also elucidate the pathophysiology of lung disease in PWCF (Figure 1).

The modulation of immune activities in a broad range by CFTR modulator therapies has been considered, albeit not extensively as one could expect [109]. Thus is because the earliest studies focused in the rescue of CFTR expression and function and mainly in cell lines and primary cells [79,110,111,112,113,114,115]. Additionally, sputum and BALF inflammatory markers have been considered mostly as a proxy of airway epithelial cells in clinical trials with CFTR modulators [116,117,118,119,120,121,122]. The appraisal of the effect of CFTR modulator therapy on the immune system, with particular reference on phagocytes, has been recently reviewed [123], with mentions of monotherapy (ivacaftor) [124,125,126,127,128,129,130,131,132], double therapy (ivacaftor/lumacaftor [129,133,134,135] and ivacaftor/tezacaftor [135,136,137]), and triple therapy (ivacaftor/tezacaftor/elexacaftor) [138]. Thus, we will highlight issues and recent investigations.

Since the introduction of CFTR modulators in the CF therapeutic toolbox, the effect of this kind of treatment on inflammation has been sparse and anecdotal. For example, one study that was conducted on 14 patients with one *G551D* allele and treated with ivacaftor did not find any alteration in sputum markers, such as free neutrophil elastase activity, α1-antitrypsin, secretory leukoprotease inhibitor, IL-1β, IL-6, and IL-8 [117]. However, in another study, it was shown that ivacaftor treatment reduced sputum levels of neutrophil elastase, IL-1β, IL-8, myeloperoxidase, and calprotectin (S100A8 and S100A9) after 1 week only, with these markers further declining over 2 years [118]. To reconcile these contrasting results, off-target drug effects can be invoked. Alternatively, differences in study design (induced vs. spontaneously expectorated sputum) and subjects’ infection and inflammatory status may be responsible. Finally, it might be that these changes may reflect an improved clinical status rather than a direct anti-inflammatory effect whether or not it was the consequence of increased CFTR function.

### 3.1. Effects of CFTR Modulators on CF Airway Epithelial Cells

Airway epithelial cells are important playmakers in the initiation and progression of CFLD since they interact directly with CF–associated bugs. However, researchers have scarcely investigated the modulation of inflammation in CF respiratory epithelial cells. Ruffin et al. demonstrated that CFTR rescue by ivacaftor/lumacaftor significantly decreased the IL-8 (CXCL8) transcription following exposure to *P. aeruginosa* diffusible material [139]. Only one study investigated monotherapies with either lumacaftor or tezacaftor, elucidating that these CFTR correctors enhanced nuclear translocations and transcriptional activation of target genes of nuclear factor E2-related factor-2 (Nrf2), a key regulator of redox balance and inflammatory signaling [140].

More recently, an unprecedented facet of the interaction of CFTR modulators with airway epithelial cells has been disclosed, i.e., inflammatory cytokines or infective agents enhance the efficacy of CFTR small-molecule drug therapies [116,141,142,143]. Rehman et al. [116] found that the treatment of a well-differentiated *F508del* homozygous airway epithelium with TNF-α+IL-17 led to a more alkalinized ASL pH, with a further increase in the presence of HEMT. Similar results were obtained with G551D-expressing CF epithelia and ivacaftor. Thus, inflammation augments ASL pH toward alkalinity and enhances the efficacy of CFTR modulators. Gentzsch et al. [142] exposed well-differentiated primary cultures of *F508del*/*F508del* human bronchial epithelia to the supernatant from mucopurulent material (SMM) harvested from the airways of excised human CF lungs or to BALF from pediatric CF patients to mimic late and early CFLD, respectively, and to double (ivacaftor/tezacaftor) or triple (ivacaftor/bamocaftor [VX-659]/lumacaftor) therapies. Both SMM and pediatric BALF enhanced *F508del* variant CFTR rescue through these combination drugs but did not alter IL-8 secretion.

Overall, these results indicate that the pre-clinical evaluation of CFTR modulator therapies should be performed in models mimicking the native airway inflammation occurring in vivo.

### 3.2. Effects of CFTR Modulators on CF Monocytes/Macrophages

The ivacaftor monotherapy normalized CD63 expression (a marker of activation and degranulation) [125]; increased levels of proteins implicated in cell migration; reduced levels of proteins involved in inflammation [126]; improved phagocytosis and M1 polarization; reduced cytokine production [129]; enhanced multiple transcriptional programs associated with inflammation, cytokine expression (TNF-α and IL-1β), and plasma levels of CCL2 (a monocyte chemokine) [130]; and, interestingly, downregulated proteins involved in leukocyte transendothelial migration and regulation of actin cytoskeleton as well as MMP-9 [131]. The dual therapy ivacaftor/lumacaftor increased the tumor suppressor and anti-inflammatory phosphatase and tensin homolog (PTEN) expression on the plasma membrane of CF monocytes [144], reduced the secretion of pro-inflammatory cytokines (IL-6, IL-8, TNF-α, IFNγ, GM-CSF) by monocyte-derived macrophages in response to *P. aeruginosa* [133], decreased *B. cenocepacia* phagocytosis [129], and reduced *Aspergillus fumigatus*-induced ROS [134].

Given the relevance of NLRP3 inflammasome-driven secretion of cytokines, Jarosz-Griffiths et al. studied the effect of different CFTR modulator regimens on the levels of IL-1β and IL-18 in serum and circulating immune cells [135]. Ivacaftor/lumacaftor attenuated IL-18 secretion through drug-naïve CF monocytes from homozygous *F508del* patients but not IL-1β, while ivacaftor/tezacaftor reduced both cytokines levels. When cytokine serum levels were studied in homozygous *F508del* patients treated with the two double combinations, it was observed that IL-18 was dramatically decreased by both ivacaftor/lumacaftor and ivacaftor/tezacaftor after 3 months of treatment, whereas IL-1β levels were diminished by ivacaftor/tezacaftor but not by ivacaftor/lumacaftor. TNF-α serum levels showed a significant decrease, an effect which was not mirrored by IL-6. Overall, a broader range of responses to ivacaftor/lumacaftor than to ivacaftor/tezacaftor was found, a phenomenon also found when peripheral blood mononuclear cells (PBMC) were studied in the same patients. An interesting downregulating effect of both oral-administered combinations was found on caspase-1 activity in mononuclear cells, while only ivacaftor/tezacafor reduced *pro-IL-1β* mRNA transcripts. Thus, there are differences in these two combination therapies, which may be related to drug pharmacokinetics or unknown factors, genetically or environmentally (i.e., infections) coded.

CF macrophages were shown to have defective lysosomal acidification and degradative function for cargoes destined to autophagosomes (*B. cenocepacia*), both improving in response to the ivacaftor/tezacaftor dual therapy [137]. In addition, ivacaftor/tezacaftor did not affect the fate of *Escherichia coli* in typical endosomes that do not acquire autophagy markers. These results appear an attempt to reconcile various contrasting results in the field about the role of CFTR in the acidification of macrophages’ organelles. Finally, it is interesting to note that ivacaftor/tezacaftor enhanced the anti-bacterial activity of (R)-roscovitine against *B. cenocepacia* and *P. aeruginosa* [136].

Regarding HEMT, Gabillard-Lefort recently reported that the triple therapy ivacaftor/tezacaftor/elexacaftor reduced the expression of the ATP P2X7 receptor with subsequently decreased NLRP3 expression, caspase-1 activation, and IL-1 secretion [138].

Two papers have recently investigated how HEMT can affect the phagocytic function of CF monocytes/macrophages. Cavinato et al. showed clinical improvement in PWCF treated with HEMT, accompanied by increased phagocytic and antimicrobial properties of CF blood-derived monocytes against *P. aeruginosa*, along with a decrease in their oxidative burst activity. The recovery of *P. aeruginosa* was partial as compared to healthy-donor monocytes and was appreciated in 10 out of 14 PWCF after 1 month and in 12 out of 17 after 6 months of treatment. Similarly, the killing activity was partially recovered in CF monocytes derived from treated patients in all PWCF after 1 month and in 8 out of 11 PWCF after 6 months of treatment. Finally, HEMT reduced IL-6 production by CF monocytes, although not significantly.

The lack of modification in cytokine secretion in response to the *B. cepacia* complex infection was a finding of Zhang et al. [145], who also showed that HEMT increased bacterial clearance by CF monocyte-derived macrophages (MDM). Interestingly, they found an increase in CFTR expression and function in MDM, although these responses varied greatly among CF individuals. Nevertheless, a 3-month post-HEMT chloride efflux correlated well with sweat chloride reductions and improvements in weight (assessed as body mass index (BMI)) and FEV_1_.

Another recently published study [146] proposes a model whereby HEMT activates MDM metabolism and enhances bacterial clearance but does not shift cells to a less-inflammatory phenotype as seen with prior modulator combinations (see, for example, Barnaby et al. [133] for ivacaftor/lumacaftor). Interestingly, in detail, they found a strong, reproducible inhibition of mitochondrial respiration by triple (HEMT) but not double CFTR modulators (ivacaftor/tezacaftor), indicating that the metabolic effect might be attributable to elexacaftor. Finally, both the modulation of phagocytosis and metabolism were operative at the level of both CF and non-CF MDM, suggesting that CFTR modulators act on these activities independently of CFTR.

Schmidt et al. [147] found that a 6-month treatment period of PWCF with HEMT did not change the circulating monocyte phenotype (as judged by markers CD10, Cd11b, CD62L) as well as the formation of platelet-monocyte complexes.

### 3.3. Effects of CFTR Modulators on Neutrophils

Several studies were conceived to investigate neutrophils phenotype and functions following CFTR modulator therapies. Thus, ivacaftor therapy partially restored degranulation mechanism via a correction of Rab27 activity [124], reduced priming/activation via normalized levels of CD11b [125], transiently increased oxidative burst via enhanced hydrogen voltage-gated channel-1 (HVCN1) expression [127], lowered activation and adhesion markers [132], and increased caveolin-1 and membrane cholesterol with consequent normalized neutrophil adhesion [128]. Ivacaftor/lumacaftor treatment was associated with significantly reduced ROS production [134].

PWCF in treatment with HEMT did not display differences in circulating neutrophils phenotypic markers (such as CD10, CD11b, CD62L, CD66b) after 6 months, although presenting clinical benefits in sweat chloride and pulmonary function [147]. ROS generation, chemotactic activity, and phagocytosis also remained unchanged, either under basal or stimulated conditions. Likewise, the formation of platelet-neutrophil complexes was comparable when analyzing PWCF before and after therapy.

Two recent studies determined that HEMT could normalize neutrophil counts after 3 or 12 months of treatment. Sheikh and colleagues [148] found a significant reduction of neutrophils at 3 months of HEMT therapy along with a reduction in plasma levels of IL-6, IL-8, and IL-17A. Dhote et al. [149] found similar results, although after 12 months of HEMT treatment in PWCF with at least one *F508del* variant and advanced lung disease. Specifically, there was a significant reduction in total leukocytes, neutrophils, monocytes, and platelets, but not in lymphocytes, within laboratory reference ranges. However, multiple-center studies are warranted to confirm functional and phenotypical changes in neutrophils associated with the clinical improvement induced by HEMT treatment.

## 4. Discussion and Conclusions

In PWCF treated with CFTR modulator therapies, there is an extreme variability in the clinical responsiveness [63,68,69,117,150]. Genetic modifiers, as presented herein, play a role in the modulation of CFLD severity and are important key players in the different patient-to-patient clinical outcomes. The limit of this review is that we have not described environmental modifiers, such as infections, which have been considered previously [151,152]. In order to be able to determine the impact of the modifier genes on the disease progression, ongoing studies need to consider crucial points: a good definition and assessment of the phenotype, causal (i.e., functional or physiological) relationships between polymorphisms and phenotype, simultaneous tests with multiple potential modifier genes, and reported associations replicable in large independent populations. A further level of analysis would be the comprehension of inflammatory/immune mechanistic pathways involving inflammation-modulating CFLD; a cross-matching of nasal epithelial cell transcriptomics and GWAS data led to the identification of human leukocyte antigen (*HLA*) and inflammatory/innate immune gene pathways in association with worse lung disease [153]. On the other hand, modifier gene studies may improve the knowledge of CF pathophysiology and lead to the development of new therapeutic interventions to reduce the consequences and increase the benefits.

Immunity in its complexity is obviously one of the targets of CFTR modulators, and herein we have presented the notion that cells and mediators of the immune system are also part of this variability. Heterogeneous results of the effects of CFTR modulators on immune cells hamper the real-life efficacy of these therapies. It is unclear whether the reported effects are drug-specific, mutation-class-specific, or depend on the degree of CFTR restoration [154]. It also could be that CFTR modulators, by normalizing the lung micro-environment, may interfere with the epigenetic reprogramming of immune cells and thus act via indirect effects [155]. Another limitation concerning the use of CFTR modulators is that no attempt was made to identify de novo effects of the modifier molecules themselves, which could be influencing off-target non-influencing CFTR effects based on diverse genetic/environmental factors [156] and be contributing to the increasing spectrum of rare side effects/toxicities being reported in a few patients [65,157]. This is also the conclusion we can draw from Zhang et al.’s data [145], who showed highly individualized responses to HEMT in MDM macrophages. Unique factors, such as gene modifiers or epigenetic modifications, might be playing a role by modifying macrophage responses to CFTR modulators [158]. Indeed, in pre/post studies, it could be difficult to understand the downstream trajectories of immune cells in a highly complex disease such as CFLD. HEMT causes numerous effects, including decreased mucus accumulation and a drop in bacterial load with subsequent indirect downstream effects on immune cell function, decreasing overall inflammation within the host and, therefore, limiting the ability to draw conclusions regarding the direct effects of modulators on immune cells. A more direct comparison between effects of vehicle, ivacaftor/tezacaftor, and HEMT in parallel in cells from the same subjects may allow us to highlight these confounding effects, as was performed by Aridgides et al. [146].

It is still to be understood if the inflammatory milieu can modify immune and airway epithelial cell response to CFTR modulators. For example, Zhang et al. [145] showed that HEMT was associated with increased phagocytic indexes in both CF and non-CF MDM. However, this effect was attenuated by exposure to CF airway supernatants. On the other hand, inflammatory cytokines and mediators found in the CF airways increased CFTR rescue by CFTR modulators in airway epithelial cells [116,142], indicating that the effect of inflammation may be cell-specific. Finally, although these recent studies have highlighted that the variable response to CFTR modulators may be due to the status of airway inflammation, the infection might also play a role since bacterial factors (e.g., pseudomonas toxins) are known to suppress CFTR expression [159,160].

Since the MDM responses correlated with clinical parameters [145], immune cell responses might be regarded as biomarkers of CFTR modulator therapies, although large, multicenter prospective studies are warranted.

In regard to gene modifiers, we did not find a cross-matching between gene variants and outcome measures of immunity during CFTR modulator therapy, although SNPs in genes that encode for immune mediators have been shown to be correlated with CFLD pathology [6,161] but only for the inflammasome. Notably, cytokines were marginally affected by the CFTR modulator therapy because of their involvement as CFLD modifier genes, thus witnessing the complexity and redundancy of the cytokine network in the lung disease pathophysiology. Nevertheless, we propose to include these gene variants (especially inflammasome) in the pre-clinical and clinical evaluation of CFTR small-molecule drug treatments.

In conclusion, the study of genetic variations in non-CFTR loci and the in-depth analysis of the effects of CFTR modulator therapies at the broad genetic level will surely enhance the understanding of inter-patient variability of CFLD and benefit the clinical outcomes of etiological small-drug CFTR therapies.

## Figures and Tables

**Table 1:** Mutation classes of the CFTR gene and molecular therapeutic interventions.

Mutation Class	Mechanism	Examples	CFTR Modulator Therapy
I	Production of a truncated CFTR mRNA → truncated protein and RNA decay	*G542X*, *R553X*, *R1162X*, *W1282X*	Read-through agents, nonsense-mediated decay pathway inhibitors, gene therapy (in development)
II	Defect in CFTR protein trafficking to the PM	*G85E*, *I507del*, *F508del*, *N1303K*	Dual combination for *F508del* homozygous or heterozygous for *F508del* and *G551D*: - potentiator (ivacaftor)/corrector (lumacaftor (Orkambi) or tezacaftor (Symdeko)) Triple combination for *F508del* heterozygous: - potentiator (ivacaftor)/correctors (tezacaftor + elexacaftor) (tradename EU: Kaftrio, tradename USA: Trikafta)
III	Channel gating defect	*S549R*, *G551D*, *G1349D*	Potentiator, e.g., ivacaftor
IV	Defect in channel conductance	*R117H*, *R334W*, *D1152H*	Potentiator, e.g., ivacaftor
V	Alternative splicing → reduction in mRNA levels	*A455E*, *2789+5G>A*, *3849+10kbC>T*	Amplifier (none in clinic)
VI	Lack of CFTR protein recycling → decrease PM levels	*F508del*, *Q1411X*	Stabilizer (none in clinic)

PM: plasma membrane.

## Data Availability

Not applicable.

## References

[1] Elborn J.S. (2016). Cystic fibrosis. Lancet.

[2] Rowe S.M., Miller S., Sorscher E.J. (2005). Cystic fibrosis. N. Engl. J. Med..

[3] Ratjen F., Bell S.C., Rowe S.M., Goss C.H., Quittner A.L., Bush A. (2015). Cystic fibrosis. Nat. Rev. Dis. Prim..

[4] Giacalone V.D., Dobosh B.S., Gaggar A., Tirouvanziam R., Margaroli C. (2020). Immunomodulation in Cystic Fibrosis: Why and How?. Int. J. Mol. Sci..

[5] Cantin A.M., Hartl D., Konstan M.W., Chmiel J.F. (2015). Inflammation in cystic fibrosis lung disease: Pathogenesis and therapy. J. Cyst. Fibros..

[6] Bruscia E.M., Bonfield T.L. (2022). Update on Innate and Adaptive Immunity in Cystic Fibrosis. Clin. Chest Med..

[7] The Cystic Fibrosis Centre at the Hospital for Sick Children (2011). Cystic Fibrosis Mutation Database.

[8] Veit G., Avramescu R.G., Chiang A.N., Houck S.A., Cai Z., Peters K.W., Hong J.S., Pollard H.B., Guggino W.B., Balch W.E. (2016). From CFTR biology toward combinatorial pharmacotherapy: Expanded classification of cystic fibrosis mutations. Mol. Biol. Cell.

[9] Boucher R.C. (2007). Airway surface dehydration in cystic fibrosis: Pathogenesis and therapy. Annu. Rev. Med..

[10] Thornton C.S., Parkins M.D. (2023). Microbial Epidemiology of the Cystic Fibrosis Airways: Past, Present, and Future. Semin. Respir. Crit. Care Med..

[11] Pezzulo A.A., Tang X.X., Hoegger M.J., Abou Alaiwa M.H., Ramachandran S., Moninger T.O., Karp P.H., Wohlford-Lenane C.L., Haagsman H.P., van Eijk M. (2012). Reduced airway surface pH impairs bacterial killing in the porcine cystic fibrosis lung. Nature.

[12] Tang X.X., Ostedgaard L.S., Hoegger M.J., Moninger T.O., Karp P.H., McMenimen J.D., Choudhury B., Varki A., Stoltz D.A., Welsh M.J. (2016). Acidic pH increases airway surface liquid viscosity in cystic fibrosis. J. Clin. Investig..

[13] Abou Alaiwa M.H., Reznikov L.R., Gansemer N.D., Sheets K.A., Horswill A.R., Stoltz D.A., Zabner J., Welsh M.J. (2014). pH modulates the activity and synergism of the airway surface liquid antimicrobials β-defensin-3 and LL-37. Proc. Natl. Acad. Sci. USA.

[14] Birket S.E., Davis J.M., Fernandez C.M., Tuggle K.L., Oden A.M., Chu K.K., Tearney G.J., Fanucchi M.V., Sorscher E.J., Rowe S.M. (2018). Development of an airway mucus defect in the cystic fibrosis rat. JCI Insight.

[15] Clary-Meinesz C., Mouroux J., Cosson J., Huitorel P., Blaive B. (1998). Influence of external pH on ciliary beat frequency in human bronchi and bronchioles. Eur. Respir. J..

[16] Cohen-Cymberknoh M., Kerem E., Ferkol T., Elizur A. (2013). Airway inflammation in cystic fibrosis: Molecular mechanisms and clinical implications. Thorax.

[17] Griese M., Kappler M., Gaggar A., Hartl D. (2008). Inhibition of airway proteases in cystic fibrosis lung disease. Eur. Respir. J..

[18] Voynow J.A., Fischer B.M., Zheng S. (2008). Proteases and cystic fibrosis. Int. J. Biochem. Cell Biol..

[19] McKelvey M.C., Weldon S., McAuley D.F., Mall M.A., Taggart C.C. (2020). Targeting Proteases in Cystic Fibrosis Lung Disease. Paradigms, Progress, and Potential. Am. J. Respir. Crit. Care Med..

[20] Cohen T.S., Prince A. (2012). Cystic fibrosis: A mucosal immunodeficiency syndrome. Nat. Med..

[21] Regamey N., Jeffery P.K., Alton E.W., Bush A., Davies J.C. (2011). Airway remodelling and its relationship to inflammation in cystic fibrosis. Thorax.

[22] Palaniyar N. (2010). Antibody equivalent molecules of the innate immune system: Parallels between innate and adaptive immune proteins. Innate Immun..

[23] Martin T.R., Frevert C.W. (2005). Innate immunity in the lungs. Proc. Am. Thorac. Soc..

[24] Nourkami-Tutdibi N., Freitag K., Zemlin M., Tutdibi E. (2021). Genetic Association with *Pseudomonas aeruginosa* Acquisition in Cystic Fibrosis: Influence of Surfactant Protein D and Mannose-Binding Lectin. Front. Immunol..

[25] Venkatakrishnan A., Stecenko A.A., King G., Blackwell T.R., Brigham K.L., Christman J.W., Blackwell T.S. (2000). Exaggerated activation of nuclear factor-kappaB and altered IkappaB-β processing in cystic fibrosis bronchial epithelial cells. Am. J. Respir. Cell Mol. Biol..

[26] Cabrini G., Rimessi A., Borgatti M., Lampronti I., Finotti A., Pinton P., Gambari R. (2020). Role of Cystic Fibrosis Bronchial Epithelium in Neutrophil Chemotaxis. Front. Immunol..

[27] De Rose V., Molloy K., Gohy S., Pilette C., Greene C.M. (2018). Airway Epithelium Dysfunction in Cystic Fibrosis and COPD. Mediat. Inflamm..

[28] Nichols D.P., Chmiel J.F. (2015). Inflammation and its genesis in cystic fibrosis. Pediatr. Pulmonol..

[29] Forrest O.A., Ingersoll S.A., Preininger M.K., Laval J., Limoli D.H., Brown M.R., Lee F.E., Bedi B., Sadikot R.T., Goldberg J.B. (2018). Frontline Science: Pathological conditioning of human neutrophils recruited to the airway milieu in cystic fibrosis. J. Leukoc. Biol..

[30] Margaroli C., Garratt L.W., Horati H., Dittrich A.S., Rosenow T., Montgomery S.T., Frey D.L., Brown M.R., Schultz C., Guglani L. (2019). Elastase Exocytosis by Airway Neutrophils Is Associated with Early Lung Damage in Children with Cystic Fibrosis. Am. J. Respir. Crit. Care Med..

[31] Turton K.B., Ingram R.J., Valvano M.A. (2021). Macrophage dysfunction in cystic fibrosis: Nature or nurture?. J. Leukoc. Biol..

[32] Koeppen K., Nymon A., Barnaby R., Li Z., Hampton T.H., Ashare A., Stanton B.A. (2021). CF monocyte-derived macrophages have an attenuated response to extracellular vesicles secreted by airway epithelial cells. Am. J. Physiol. Lung Cell. Mol. Physiol..

[33] Bruscia E.M., Zhang P.X., Ferreira E., Caputo C., Emerson J.W., Tuck D., Krause D.S., Egan M.E. (2009). Macrophages directly contribute to the exaggerated inflammatory response in cystic fibrosis transmembrane conductance regulator^−/−^ mice. Am. J. Respir. Cell Mol. Biol..

[34] Di A., Brown M.E., Deriy L.V., Li C., Szeto F.L., Chen Y., Huang P., Tong J., Naren A.P., Bindokas V. (2006). CFTR regulates phagosome acidification in macrophages and alters bactericidal activity. Nat. Cell Biol..

[35] Averna M., Bavestrello M., Cresta F., Pedrazzi M., De Tullio R., Minicucci L., Sparatore B., Salamino F., Pontremoli S., Melloni E. (2016). Abnormal activation of calpain and protein kinase Calpha promotes a constitutive release of matrix metalloproteinase 9 in peripheral blood mononuclear cells from cystic fibrosis patients. Arch. Biochem. Biophys..

[36] Haggie P.M., Verkman A.S. (2007). Cystic fibrosis transmembrane conductance regulator-independent phagosomal acidification in macrophages. J. Biol. Chem..

[37] Barriere H., Bagdany M., Bossard F., Okiyoneda T., Wojewodka G., Gruenert D., Radzioch D., Lukacs G.L. (2009). Revisiting the role of cystic fibrosis transmembrane conductance regulator and counterion permeability in the pH regulation of endocytic organelles. Mol. Biol. Cell.

[38] Law S.M., Stanfield S.J., Hardisty G.R., Dransfield I., Campbell C.J., Gray R.D. (2020). Human cystic fibrosis monocyte derived macrophages display no defect in acidification of phagolysosomes when measured by optical nanosensors. J. Cyst. Fibros..

[39] McElvaney O.J., Zaslona Z., Becker-Flegler K., Palsson-McDermott E.M., Boland F., Gunaratnam C., Gulbins E., O’Neill L.A., Reeves E.P., McElvaney N.G. (2019). Specific Inhibition of the NLRP3 Inflammasome as an Antiinflammatory Strategy in Cystic Fibrosis. Am. J. Respir. Crit. Care Med..

[40] Schupp J.C., Khanal S., Gomez J.L., Sauler M., Adams T.S., Chupp G.L., Yan X., Poli S., Zhao Y., Montgomery R.R. (2020). Single-Cell Transcriptional Archetypes of Airway Inflammation in Cystic Fibrosis. Am. J. Respir. Crit. Care Med..

[41] Vencken S.F., Greene C.M. (2016). Toll-Like Receptors in Cystic Fibrosis: Impact of Dysfunctional microRNA on Innate Immune Responses in the Cystic Fibrosis Lung. J. Innate Immun..

[42] Foell D., Seeliger S., Vogl T., Koch H.G., Maschek H., Harms E., Sorg C., Roth J. (2003). Expression of S100A12 (EN-RAGE) in cystic fibrosis. Thorax.

[43] Entezari M., Weiss D.J., Sitapara R., Whittaker L., Wargo M.J., Li J., Wang H., Yang H., Sharma L., Phan B.D. (2012). Inhibition of high-mobility group box 1 protein (HMGB1) enhances bacterial clearance and protects against *Pseudomonas aeruginosa* pneumonia in cystic fibrosis. Mol. Med..

[44] Rowe S.M., Jackson P.L., Liu G., Hardison M., Livraghi A., Solomon G.M., McQuaid D.B., Noerager B.D., Gaggar A., Clancy J.P. (2008). Potential role of high-mobility group box 1 in cystic fibrosis airway disease. Am. J. Respir. Crit. Care Med..

[45] Hunt W.R., Helfman B.R., McCarty N.A., Hansen J.M. (2016). Advanced glycation end products are elevated in cystic fibrosis-related diabetes and correlate with worse lung function. J. Cyst. Fibros..

[46] Lara-Reyna S., Holbrook J., Jarosz-Griffiths H.H., Peckham D., McDermott M.F. (2020). Dysregulated signalling pathways in innate immune cells with cystic fibrosis mutations. Cell. Mol. Life Sci..

[47] Scambler T., Jarosz-Griffiths H.H., Lara-Reyna S., Pathak S., Wong C., Holbrook J., Martinon F., Savic S., Peckham D., McDermott M.F. (2019). ENaC-mediated sodium influx exacerbates NLRP3-dependent inflammation in cystic fibrosis. eLife.

[48] Balazs A., Mall M.A. (2019). Mucus obstruction and inflammation in early cystic fibrosis lung disease: Emerging role of the IL-1 signaling pathway. Pediatr. Pulmonol..

[49] Iannitti R.G., Napolioni V., Oikonomou V., De Luca A., Galosi C., Pariano M., Massi-Benedetti C., Borghi M., Puccetti M., Lucidi V. (2016). IL-1 receptor antagonist ameliorates inflammasome-dependent inflammation in murine and human cystic fibrosis. Nat. Commun..

[50] Lara-Reyna S., Scambler T., Holbrook J., Wong C., Jarosz-Griffiths H.H., Martinon F., Savic S., Peckham D., McDermott M.F. (2019). Metabolic Reprograming of Cystic Fibrosis Macrophages via the IRE1alpha Arm of the Unfolded Protein Response Results in Exacerbated Inflammation. Front. Immunol..

[51] Montgomery S.T., Dittrich A.S., Garratt L.W., Turkovic L., Frey D.L., Stick S.M., Mall M.A., Kicic A., Arest C.F. (2018). Interleukin-1 is associated with inflammation and structural lung disease in young children with cystic fibrosis. J. Cyst. Fibros..

[52] Rao S., Grigg J. (2006). New insights into pulmonary inflammation in cystic fibrosis. Arch. Dis. Child..

[53] Montgomery S.T., Mall M.A., Kicic A., Stick S.M., Arest C.F. (2017). Hypoxia and sterile inflammation in cystic fibrosis airways: Mechanisms and potential therapies. Eur. Respir. J..

[54] Keiser N.W., Birket S.E., Evans I.A., Tyler S.R., Crooke A.K., Sun X., Zhou W., Nellis J.R., Stroebele E.K., Chu K.K. (2015). Defective innate immunity and hyperinflammation in newborn cystic fibrosis transmembrane conductance regulator-knockout ferret lungs. Am. J. Respir. Cell Mol. Biol..

[55] Rosenow T., Mok L.C., Turkovic L., Berry L.J., Sly P.D., Ranganathan S., Tiddens H., Stick S.M. (2019). The cumulative effect of inflammation and infection on structural lung disease in early cystic fibrosis. Eur. Respir. J..

[56] Polverino F., Lu B., Quintero J.R., Vargas S.O., Patel A.S., Owen C.A., Gerard N.P., Gerard C., Cernadas M. (2019). CFTR regulates B cell activation and lymphoid follicle development. Respir. Res..

[57] Hector A., Schafer H., Poschel S., Fischer A., Fritzsching B., Ralhan A., Carevic M., Oz H., Zundel S., Hogardt M. (2015). Regulatory T-cell impairment in cystic fibrosis patients with chronic pseudomonas infection. Am. J. Respir. Crit. Care Med..

[58] Tan H.L., Regamey N., Brown S., Bush A., Lloyd C.M., Davies J.C. (2011). The Th17 pathway in cystic fibrosis lung disease. Am. J. Respir. Crit. Care Med..

[59] Dubin P.J., Kolls J.K. (2011). IL-17 in cystic fibrosis: More than just Th17 cells. Am. J. Respir. Crit. Care Med..

[60] Hsu D., Taylor P., Fletcher D., van Heeckeren R., Eastman J., van Heeckeren A., Davis P., Chmiel J.F., Pearlman E., Bonfield T.L. (2016). Interleukin-17 Pathophysiology and Therapeutic Intervention in Cystic Fibrosis Lung Infection and Inflammation. Infect. Immun..

[61] Decraene A., Willems-Widyastuti A., Kasran A., De Boeck K., Bullens D.M., Dupont L.J. (2010). Elevated expression of both mRNA and protein levels of IL-17A in sputum of stable Cystic Fibrosis patients. Respir. Res..

[62] Tan H.L., Rosenthal M. (2013). IL-17 in lung disease: Friend or foe?. Thorax.

[63] Ramsey B.W., Davies J., McElvaney N.G., Tullis E., Bell S.C., Drevinek P., Griese M., McKone E.F., Wainwright C.E., Konstan M.W. (2011). A CFTR potentiator in patients with cystic fibrosis and the G551D mutation. N. Engl. J. Med..

[64] Jia S., Taylor-Cousar J.L. (2023). Cystic Fibrosis Modulator Therapies. Annu. Rev. Med..

[65] Southern K.W., Castellani C., Lammertyn E., Smyth A., VanDevanter D., van Koningsbruggen-Rietschel S., Barben J., Bevan A., Brokaar E., Collins S. (2023). Standards of care for CFTR variant-specific therapy (including modulators) for people with cystic fibrosis. J. Cyst. Fibros..

[66] Taylor-Cousar J.L., Robinson P.D., Shteinberg M., Downey D.G. (2023). CFTR modulator therapy: Transforming the landscape of clinical care in cystic fibrosis. Lancet.

[67] Skilton M., Krishan A., Patel S., Sinha I.P., Southern K.W. (2019). Potentiators (specific therapies for class III and IV mutations) for cystic fibrosis. Cochrane Database Syst. Rev..

[68] Donaldson S.H., Pilewski J.M., Griese M., Cooke J., Viswanathan L., Tullis E., Davies J.C., Lekstrom-Himes J.A., Wang L.T., Group V.X.S. (2018). Tezacaftor/Ivacaftor in Subjects with Cystic Fibrosis and F508del/F508del-CFTR or F508del/G551D-CFTR. Am. J. Respir. Crit. Care Med..

[69] Wainwright C.E., Elborn J.S., Ramsey B.W., Marigowda G., Huang X., Cipolli M., Colombo C., Davies J.C., De Boeck K., Flume P.A. (2015). Lumacaftor-Ivacaftor in Patients with Cystic Fibrosis Homozygous for Phe508del CFTR. N. Engl. J. Med..

[70] Taylor-Cousar J.L., Munck A., McKone E.F., van der Ent C.K., Moeller A., Simard C., Wang L.T., Ingenito E.P., McKee C., Lu Y. (2017). Tezacaftor-Ivacaftor in Patients with Cystic Fibrosis Homozygous for Phe508del. N. Engl. J. Med..

[71] Rowe S.M., Daines C., Ringshausen F.C., Kerem E., Wilson J., Tullis E., Nair N., Simard C., Han L., Ingenito E.P. (2017). Tezacaftor-Ivacaftor in Residual-Function Heterozygotes with Cystic Fibrosis. N. Engl. J. Med..

[72] Heijerman H.G.M., McKone E.F., Downey D.G., Van Braeckel E., Rowe S.M., Tullis E., Mall M.A., Welter J.J., Ramsey B.W., McKee C.M. (2019). Efficacy and safety of the elexacaftor plus tezacaftor plus ivacaftor combination regimen in people with cystic fibrosis homozygous for the F508del mutation: A double-blind, randomised, phase 3 trial. Lancet.

[73] Middleton P.G., Mall M.A., Drevinek P., Lands L.C., McKone E.F., Polineni D., Ramsey B.W., Taylor-Cousar J.L., Tullis E., Vermeulen F. (2019). Elexacaftor-Tezacaftor-Ivacaftor for Cystic Fibrosis with a Single Phe508del Allele. N. Engl. J. Med..

[74] Pranke I., Golec A., Hinzpeter A., Edelman A., Sermet-Gaudelus I. (2019). Emerging Therapeutic Approaches for Cystic Fibrosis. From Gene Editing to Personalized Medicine. Front. Pharmacol..

[75] Sepahzad A., Morris-Rosendahl D.J., Davies J.C. (2021). Cystic Fibrosis Lung Disease Modifiers and Their Relevance in the New Era of Precision Medicine. Genes.

[76] Drumm M.L., Konstan M.W., Schluchter M.D., Handler A., Pace R., Zou F., Zariwala M., Fargo D., Xu A., Dunn J.M. (2005). Genetic modifiers of lung disease in cystic fibrosis. N. Engl. J. Med..

[77] Eckford P.D., Ramjeesingh M., Molinski S., Pasyk S., Dekkers J.F., Li C., Ahmadi S., Ip W., Chung T.E., Du K. (2014). VX-809 and related corrector compounds exhibit secondary activity stabilizing active F508del-CFTR after its partial rescue to the cell surface. Chem. Biol..

[78] Dekkers J.F., Berkers G., Kruisselbrink E., Vonk A., de Jonge H.R., Janssens H.M., Bronsveld I., van de Graaf E.A., Nieuwenhuis E.E., Houwen R.H. (2016). Characterizing responses to CFTR-modulating drugs using rectal organoids derived from subjects with cystic fibrosis. Sci. Transl. Med..

[79] Pranke I.M., Hatton A., Simonin J., Jais J.P., Le Pimpec-Barthes F., Carsin A., Bonnette P., Fayon M., Stremler-Le Bel N., Grenet D. (2017). Correction of CFTR function in nasal epithelial cells from cystic fibrosis patients predicts improvement of respiratory function by CFTR modulators. Sci. Rep..

[80] Bacalhau M., Camargo M., Magalhaes-Ghiotto G.A.V., Drumond S., Castelletti C.H.M., Lopes-Pacheco M. (2023). Elexacaftor-Tezacaftor-Ivacaftor: A Life-Changing Triple Combination of CFTR Modulator Drugs for Cystic Fibrosis. Pharmaceuticals.

[81] Mesinele J., Ruffin M., Guillot L., Corvol H. (2022). Modifier Factors of Cystic Fibrosis Phenotypes: A Focus on Modifier Genes. Int. J. Mol. Sci..

[82] Slieker M.G., Sanders E.A., Rijkers G.T., Ruven H.J., van der Ent C.K. (2005). Disease modifying genes in cystic fibrosis. J. Cyst. Fibros..

[83] Guillot L., Beucher J., Tabary O., Le Rouzic P., Clement A., Corvol H. (2014). Lung disease modifier genes in cystic fibrosis. Int. J. Biochem. Cell Biol..

[84] Kuroki Y., Takahashi M., Nishitani C. (2007). Pulmonary collectins in innate immunity of the lung. Cell. Microbiol..

[85] Garred P., Pressler T., Madsen H.O., Frederiksen B., Svejgaard A., Hoiby N., Schwartz M., Koch C. (1999). Association of mannose-binding lectin gene heterogeneity with severity of lung disease and survival in cystic fibrosis. J. Clin. Investig..

[86] Noah T.L., Murphy P.C., Alink J.J., Leigh M.W., Hull W.M., Stahlman M.T., Whitsett J.A. (2003). Bronchoalveolar lavage fluid surfactant protein-A and surfactant protein-D are inversely related to inflammation in early cystic fibrosis. Am. J. Respir. Crit. Care Med..

[87] Dorfman R., Sandford A., Taylor C., Huang B., Frangolias D., Wang Y., Sang R., Pereira L., Sun L., Berthiaume Y. (2008). Complex two-gene modulation of lung disease severity in children with cystic fibrosis. J. Clin. Investig..

[88] Zuo L., Wang K., Luo X. (2014). Use of diplotypes—matched haplotype pairs from homologous chromosomes—in gene-disease association studies. Shanghai Arch. Psychiatry.

[89] Lin Z., Thorenoor N., Wu R., DiAngelo S.L., Ye M., Thomas N.J., Liao X., Lin T.R., Warren S., Floros J. (2018). Genetic Association of Pulmonary Surfactant Protein Genes, SFTPA1, SFTPA2, SFTPB, SFTPC, and SFTPD With Cystic Fibrosis. Front. Immunol..

[90] Greene C.M., Carroll T.P., Smith S.G., Taggart C.C., Devaney J., Griffin S., O’Neill S.J., McElvaney N.G. (2005). TLR-induced inflammation in cystic fibrosis and non-cystic fibrosis airway epithelial cells. J. Immunol..

[91] Haerynck F., Mahachie John J.M., Van Steen K., Schelstraete P., Van daele S., Loeys B., Van Thielen M., De Canck I., Nuytinck L., De Baets F. (2013). Genetic variations in toll-like receptor pathway and lung function decline in Cystic fibrosis patients. Hum. Immunol..

[92] Blohmke C.J., Park J., Hirschfeld A.F., Victor R.E., Schneiderman J., Stefanowicz D., Chilvers M.A., Durie P.R., Corey M., Zielenski J. (2010). TLR5 as an anti-inflammatory target and modifier gene in cystic fibrosis. J. Immunol..

[93] Beucher J., Boelle P.Y., Busson P.F., Muselet-Charlier C., Clement A., Corvol H., The French C F Modifier Gene Study Investigators (2012). AGER−429T/C is associated with an increased lung disease severity in cystic fibrosis. PLoS ONE.

[94] De Torre-Minguela C., Mesa Del Castillo P., Pelegrin P. (2017). The NLRP3 and Pyrin Inflammasomes: Implications in the Pathophysiology of Autoinflammatory Diseases. Front. Immunol..

[95] Atalay M., Şen B., Dayangaç Erden D. (2023). NLRP3 inflammasome as a novel target for cystic fibrosis treatment. Bull. Natl. Res. Cent..

[96] Graustein A.D., Berrington W.R., Buckingham K.J., Nguyen F.K., Joudeh L.L., Rosenfeld M., Bamshad M.J., Gibson R.L., Hawn T.R., Emond M.J. (2021). Inflammasome Genetic Variants, Macrophage Function, and Clinical Outcomes in Cystic Fibrosis. Am. J. Respir. Cell Mol. Biol..

[97] Guan X., Hou Y., Sun F., Yang Z., Li C. (2016). Dysregulated Chemokine Signaling in Cystic Fibrosis Lung Disease: A Potential Therapeutic Target. Curr. Drug Targets.

[98] Hillian A.D., Londono D., Dunn J.M., Goddard K.A., Pace R.G., Knowles M.R., Drumm M.L., CF Gene Modifier Study Group (2008). Modulation of cystic fibrosis lung disease by variants in interleukin-8. Genes Immun..

[99] De Vries L., Griffiths A., Armstrong D., Robinson P.J. (2014). Cytokine gene polymorphisms and severity of CF lung disease. J. Cyst. Fibros..

[100] Stanke F., Becker T., Kumar V., Hedtfeld S., Becker C., Cuppens H., Tamm S., Yarden J., Laabs U., Siebert B. (2011). Genes that determine immunology and inflammation modify the basic defect of impaired ion conductance in cystic fibrosis epithelia. J. Med. Genet..

[101] Labenski H., Hedtfeld S., Becker T., Tummler B., Stanke F. (2011). Initial interrogation, confirmation and fine mapping of modifying genes: STAT3, IL1B and IFNGR1 determine cystic fibrosis disease manifestation. Eur. J. Hum. Genet..

[102] Locksley R.M., Killeen N., Lenardo M.J. (2001). The TNF and TNF receptor superfamilies: Integrating mammalian biology. Cell.

[103] Shmarina G., Pukhalsky A., Petrova N., Zakharova E., Avakian L., Kapranov N., Alioshkin V. (2013). TNF gene polymorphisms in cystic fibrosis patients: Contribution to the disease progression. J. Transl. Med..

[104] Sagwal S., Chauhan A., Kaur J., Prasad R., Singh M., Singh M. (2020). Association of Serum TGF-beta1 Levels with Different Clinical Phenotypes of Cystic Fibrosis Exacerbation. Lung.

[105] Corvol H., Boelle P.Y., Brouard J., Knauer N., Chadelat K., Henrion-Caude A., Flamant C., Muselet-Charlier C., Boule M., Fauroux B. (2008). Genetic variations in inflammatory mediators influence lung disease progression in cystic fibrosis. Pediatr. Pulmonol..

[106] Trojan T., Alejandre Alcazar M.A., Fink G., Thomassen J.C., Maessenhausen M.V., Rietschel E., Schneider P.M., van Koningsbruggen-Rietschel S. (2022). The effect of TGF-β(1) polymorphisms on pulmonary disease progression in patients with cystic fibrosis. BMC Pulm. Med..

[107] Furlan L.L., Marson F.A., Ribeiro J.D., Bertuzzo C.S., Salomao Junior J.B., Souza D.R. (2016). IL8 gene as modifier of cystic fibrosis: Unraveling the factors which influence clinical variability. Hum. Genet..

[108] Hassanzad M., Farnia P., Ghanavi J., Parvini F., Saif S., Velayati A.A. (2019). TNFalpha −857 C/T and TNFR2 +587 T/G polymorphisms are associated with cystic fibrosis in Iranian patients. Eur. J. Med. Genet..

[109] Keown K., Brown R., Doherty D.F., Houston C., McKelvey M.C., Creane S., Linden D., McAuley D.F., Kidney J.C., Weldon S. (2020). Airway Inflammation and Host Responses in the Era of CFTR Modulators. Int. J. Mol. Sci..

[110] Van Goor F., Hadida S., Grootenhuis P.D., Burton B., Cao D., Neuberger T., Turnbull A., Singh A., Joubran J., Hazlewood A. (2009). Rescue of CF airway epithelial cell function in vitro by a CFTR potentiator, VX-770. Proc. Natl. Acad. Sci. USA.

[111] Awatade N.T., Uliyakina I., Farinha C.M., Clarke L.A., Mendes K., Sole A., Pastor J., Ramos M.M., Amaral M.D. (2015). Measurements of Functional Responses in Human Primary Lung Cells as a Basis for Personalized Therapy for Cystic Fibrosis. EBioMedicine.

[112] Awatade N.T., Wong S.L., Hewson C.K., Fawcett L.K., Kicic A., Jaffe A., Waters S.A. (2018). Human Primary Epithelial Cell Models: Promising Tools in the Era of Cystic Fibrosis Personalized Medicine. Front. Pharmacol..

[113] Van Goor F., Hadida S., Grootenhuis P.D., Burton B., Stack J.H., Straley K.S., Decker C.J., Miller M., McCartney J., Olson E.R. (2011). Correction of the F508del-CFTR protein processing defect in vitro by the investigational drug VX-809. Proc. Natl. Acad. Sci. USA.

[114] Keating D., Marigowda G., Burr L., Daines C., Mall M.A., McKone E.F., Ramsey B.W., Rowe S.M., Sass L.A., Tullis E. (2018). VX-445-Tezacaftor-Ivacaftor in Patients with Cystic Fibrosis and One or Two Phe508del Alleles. N. Engl. J. Med..

[115] Davies J.C., Moskowitz S.M., Brown C., Horsley A., Mall M.A., McKone E.F., Plant B.J., Prais D., Ramsey B.W., Taylor-Cousar J.L. (2018). VX-659-Tezacaftor-Ivacaftor in Patients with Cystic Fibrosis and One or Two Phe508del Alleles. N. Engl. J. Med..

[116] Rehman T., Karp P.H., Tan P., Goodell B.J., Pezzulo A.A., Thurman A.L., Thornell I.M., Durfey S.L., Duffey M.E., Stoltz D.A. (2021). Inflammatory cytokines TNF-α and IL-17 enhance the efficacy of cystic fibrosis transmembrane conductance regulator modulators. J. Clin. Investig..

[117] Rowe S.M., Heltshe S.L., Gonska T., Donaldson S.H., Borowitz D., Gelfond D., Sagel S.D., Khan U., Mayer-Hamblett N., Van Dalfsen J.M. (2014). Clinical mechanism of the cystic fibrosis transmembrane conductance regulator potentiator ivacaftor in G551D-mediated cystic fibrosis. Am. J. Respir. Crit. Care Med..

[118] Hisert K.B., Heltshe S.L., Pope C., Jorth P., Wu X., Edwards R.M., Radey M., Accurso F.J., Wolter D.J., Cooke G. (2017). Restoring Cystic Fibrosis Transmembrane Conductance Regulator Function Reduces Airway Bacteria and Inflammation in People with Cystic Fibrosis and Chronic Lung Infections. Am. J. Respir. Crit. Care Med..

[119] Harris J.K., Wagner B.D., Zemanick E.T., Robertson C.E., Stevens M.J., Heltshe S.L., Rowe S.M., Sagel S.D. (2020). Changes in Airway Microbiome and Inflammation with Ivacaftor Treatment in Patients with Cystic Fibrosis and the G551D Mutation. Ann. Am. Thorac. Soc..

[120] McNally P., Butler D., Karpievitch Y.V., Linnane B., Ranganathan S., Stick S.M., Hall G.L., Schultz A. (2021). Ivacaftor and Airway Inflammation in Preschool Children with Cystic Fibrosis. Am. J. Respir. Crit. Care Med..

[121] Mainz J.G., Arnold C., Wittstock K., Hipler U.C., Lehmann T., Zagoya C., Duckstein F., Ellemunter H., Hentschel J. (2021). Ivacaftor Reduces Inflammatory Mediators in Upper Airway Lining Fluid from Cystic Fibrosis Patients with a G551D Mutation: Serial Non-Invasive Home-Based Collection of Upper Airway Lining Fluid. Front. Immunol..

[122] Graeber S.Y., Boutin S., Wielputz M.O., Joachim C., Frey D.L., Wege S., Sommerburg O., Kauczor H.U., Stahl M., Dalpke A.H. (2021). Effects of Lumacaftor-Ivacaftor on Lung Clearance Index, Magnetic Resonance Imaging, and Airway Microbiome in Phe508del Homozygous Patients with Cystic Fibrosis. Ann. Am. Thorac. Soc..

[123] Meoli A., Eickmeier O., Pisi G., Fainardi V., Zielen S., Esposito S. (2022). Impact of CFTR Modulators on the Impaired Function of Phagocytes in Cystic Fibrosis Lung Disease. Int. J. Mol. Sci..

[124] Pohl K., Hayes E., Keenan J., Henry M., Meleady P., Molloy K., Jundi B., Bergin D.A., McCarthy C., McElvaney O.J. (2014). A neutrophil intrinsic impairment affecting Rab27a and degranulation in cystic fibrosis is corrected by CFTR potentiator therapy. Blood.

[125] Bratcher P.E., Rowe S.M., Reeves G., Roberts T., Szul T., Harris W.T., Tirouvanziam R., Gaggar A. (2016). Alterations in blood leukocytes of G551D-bearing cystic fibrosis patients undergoing treatment with ivacaftor. J. Cyst. Fibros..

[126] Hisert K.B., Schoenfelt K.Q., Cooke G., Grogan B., Launspach J.L., Gallagher C.G., Donnelly S.C., Welsh M.J., Singh P.K., McKone E.F. (2016). Ivacaftor-Induced Proteomic Changes Suggest Monocyte Defects May Contribute to the Pathogenesis of Cystic Fibrosis. Am. J. Respir. Cell Mol. Biol..

[127] Guerra L., D’Oria S., Favia M., Castellani S., Santostasi T., Polizzi A.M., Mariggio M.A., Gallo C., Casavola V., Montemurro P. (2017). CFTR-dependent chloride efflux in cystic fibrosis mononuclear cells is increased by ivacaftor therapy. Pediatr. Pulmonol..

[128] White M.M., Geraghty P., Hayes E., Cox S., Leitch W., Alfawaz B., Lavelle G.M., McElvaney O.J., Flannery R., Keenan J. (2017). Neutrophil Membrane Cholesterol Content is a Key Factor in Cystic Fibrosis Lung Disease. EBioMedicine.

[129] Zhang S., Shrestha C.L., Kopp B.T. (2018). Cystic fibrosis transmembrane conductance regulator (CFTR) modulators have differential effects on cystic fibrosis macrophage function. Sci. Rep..

[130] Hisert K.B., Birkland T.P., Schoenfelt K.Q., Long M.E., Grogan B., Carter S., Liles W.C., McKone E.F., Becker L., Manicone A.M. (2020). CFTR Modulator Therapy Enhances Peripheral Blood Monocyte Contributions to Immune Responses in People with Cystic Fibrosis. Front. Pharmacol..

[131] Pedrazzi M., Vercellone S., Barberis E., Capraro M., De Tullio R., Cresta F., Casciaro R., Castellani C., Patrone M., Marengo E. (2021). Identification of Potential Leukocyte Biomarkers Related to Drug Recovery of CFTR: Clinical Applications in Cystic Fibrosis. Int. J. Mol. Sci..

[132] Hardisty G.R., Law S.M., Carter S., Grogan B., Singh P.K., McKone E.F., Gray R.D. (2021). Ivacaftor modifies cystic fibrosis neutrophil phenotype in subjects with R117H residual function CFTR mutations. Eur. Respir. J..

[133] Barnaby R., Koeppen K., Nymon A., Hampton T.H., Berwin B., Ashare A., Stanton B.A. (2018). Lumacaftor (VX-809) restores the ability of CF macrophages to phagocytose and kill *Pseudomonas aeruginosa*. Am. J. Physiol. Lung Cell. Mol. Physiol..

[134] Currie A.J., Main E.T., Wilson H.M., Armstrong-James D., Warris A. (2020). CFTR Modulators Dampen Aspergillus-Induced Reactive Oxygen Species Production by Cystic Fibrosis Phagocytes. Front. Cell. Infect. Microbiol..

[135] Jarosz-Griffiths H.H., Scambler T., Wong C.H., Lara-Reyna S., Holbrook J., Martinon F., Savic S., Whitaker P., Etherington C., Spoletini G. (2020). Different CFTR modulator combinations downregulate inflammation differently in cystic fibrosis. eLife.

[136] Shrestha C.L., Zhang S., Wisniewski B., Hafner S., Elie J., Meijer L., Kopp B.T. (2020). (R)-Roscovitine and CFTR modulators enhance killing of multi-drug resistant *Burkholderia cenocepacia* by cystic fibrosis macrophages. Sci. Rep..

[137] Badr A., Eltobgy M., Krause K., Hamilton K., Estfanous S., Daily K.P., Abu Khweek A., Hegazi A., Anne M.N.K., Carafice C. (2022). CFTR Modulators Restore Acidification of Autophago-Lysosomes and Bacterial Clearance in Cystic Fibrosis Macrophages. Front. Cell. Infect. Microbiol..

[138] Gabillard-Lefort C., Casey M., Glasgow A.M.A., Boland F., Kerr O., Marron E., Lyons A.M., Gunaratnam C., McElvaney N.G., Reeves E.P. (2022). Trikafta Rescues CFTR and Lowers Monocyte P2X7R-induced Inflammasome Activation in Cystic Fibrosis. Am. J. Respir. Crit. Care Med..

[139] Ruffin M., Roussel L., Maille E., Rousseau S., Brochiero E. (2018). Vx-809/Vx-770 treatment reduces inflammatory response to *Pseudomonas aeruginosa* in primary differentiated cystic fibrosis bronchial epithelial cells. Am. J. Physiol. Lung Cell. Mol. Physiol..

[140] Borcherding D.C., Siefert M.E., Lin S., Brewington J., Sadek H., Clancy J.P., Plafker S.M., Ziady A.G. (2019). Clinically-approved CFTR modulators rescue Nrf2 dysfunction in cystic fibrosis airway epithelia. J. Clin. Investig..

[141] Ribeiro C.M.P., Gentzsch M. (2021). Impact of Airway Inflammation on the Efficacy of CFTR Modulators. Cells.

[142] Gentzsch M., Cholon D.M., Quinney N.L., Martino M.E.B., Minges J.T., Boyles S.E., Guhr Lee T.N., Esther C.R., Ribeiro C.M.P. (2021). Airway Epithelial Inflammation In Vitro Augments the Rescue of Mutant CFTR by Current CFTR Modulator Therapies. Front. Pharmacol..

[143] Rehman T., Welsh M.J. (2023). Inflammation as a Regulator of the Airway Surface Liquid pH in Cystic Fibrosis. Cells.

[144] Riquelme S.A., Hopkins B.D., Wolfe A.L., DiMango E., Kitur K., Parsons R., Prince A. (2017). Cystic Fibrosis Transmembrane Conductance Regulator Attaches Tumor Suppressor PTEN to the Membrane and Promotes Anti *Pseudomonas aeruginosa* Immunity. Immunity.

[145] Zhang S., Shrestha C.L., Robledo-Avila F., Jaganathan D., Wisniewski B.L., Brown N., Pham H., Carey K., Amer A.O., Hall-Stoodley L. (2023). Cystic fibrosis macrophage function and clinical outcomes after elexacaftor/tezacaftor/ivacaftor. Eur. Respir. J..

[146] Aridgides D.S., Mellinger D.L., Gwilt L.L., Hampton T.H., Mould D.L., Hogan D.A., Ashare A. (2023). Comparative effects of CFTR modulators on phagocytic, metabolic and inflammatory profiles of CF and nonCF macrophages. Sci. Rep..

[147] Schmidt H., Hopfer L.M., Wohlgemuth L., Knapp C.L., Mohamed A.O.K., Stukan L., Munnich F., Husken D., Koller A.S., Stratmann A.E.P. (2023). Multimodal analysis of granulocytes, monocytes, and platelets in patients with cystic fibrosis before and after Elexacaftor-Tezacaftor-Ivacaftor treatment. Front. Immunol..

[148] Sheikh S., Britt R.D., Ryan-Wenger N.A., Khan A.Q., Lewis B.W., Gushue C., Ozuna H., Jaganathan D., McCoy K., Kopp B.T. (2023). Impact of elexacaftor-tezacaftor-ivacaftor on bacterial colonization and inflammatory responses in cystic fibrosis. Pediatr. Pulmonol..

[149] Dhote T., Martin C., Regard L., Pesenti L., Kanaan R., Carlier N., Honore I., Da Silva J., Witko-Sarsat V., Burgel P.R. (2023). Normalisation of circulating neutrophil counts after 12 months of elexacaftor-tezacaftor-ivacaftor in patients with advanced cystic fibrosis. Eur. Respir. J..

[150] Boyle M.P., Bell S.C., Konstan M.W., McColley S.A., Rowe S.M., Rietschel E., Huang X., Waltz D., Patel N.R., Rodman D. (2014). A CFTR corrector (lumacaftor) and a CFTR potentiator (ivacaftor) for treatment of patients with cystic fibrosis who have a phe508del CFTR mutation: A phase 2 randomised controlled trial. Lancet Respir. Med..

[151] Bhagirath A.Y., Li Y., Somayajula D., Dadashi M., Badr S., Duan K. (2016). Cystic fibrosis lung environment and *Pseudomonas aeruginosa* infection. BMC Pulm. Med..

[152] Hampton T.H., Thomas D., van der Gast C., O’Toole G.A., Stanton B.A. (2021). Mild Cystic Fibrosis Lung Disease Is Associated with Bacterial Community Stability. Microbiol. Spectr..

[153] Polineni D., Dang H., Gallins P.J., Jones L.C., Pace R.G., Stonebraker J.R., Commander L.A., Krenicky J.E., Zhou Y.H., Corvol H. (2018). Airway Mucosal Host Defense Is Key to Genomic Regulation of Cystic Fibrosis Lung Disease Severity. Am. J. Respir. Crit. Care Med..

[154] Sergeev V., Chou F.Y., Lam G.Y., Hamilton C.M., Wilcox P.G., Quon B.S. (2020). The Extrapulmonary Effects of Cystic Fibrosis Transmembrane Conductance Regulator Modulators in Cystic Fibrosis. Ann. Am. Thorac. Soc..

[155] Hey J., Paulsen M., Toth R., Weichenhan D., Butz S., Schatterny J., Liebers R., Lutsik P., Plass C., Mall M.A. (2021). Epigenetic reprogramming of airway macrophages promotes polarization and inflammation in muco-obstructive lung disease. Nat. Commun..

[156] Butnariu L.I., Tarca E., Cojocaru E., Rusu C., Moisa S.M., Leon Constantin M.M., Gorduza E.V., Trandafir L.M. (2021). Genetic Modifying Factors of Cystic Fibrosis Phenotype: A Challenge for Modern Medicine. J. Clin. Med..

[157] Dagenais S., Russo L., Madsen A., Webster J., Becnel L. (2022). Use of Real-World Evidence to Drive Drug Development Strategy and Inform Clinical Trial Design. Clin. Pharmacol. Ther..

[158] Lopes-Pacheco M. (2020). CFTR Modulators: The Changing Face of Cystic Fibrosis in the Era of Precision Medicine. Front. Pharmacol..

[159] Bomberger J.M., Ye S., Maceachran D.P., Koeppen K., Barnaby R.L., O’Toole G.A., Stanton B.A. (2011). A *Pseudomonas aeruginosa* toxin that hijacks the host ubiquitin proteolytic system. PLoS Pathog..

[160] Stanton B.A. (2017). Effects of *Pseudomonas aeruginosa* on CFTR chloride secretion and the host immune response. Am. J. Physiol. Cell Physiol..

[161] Joynt A.T., Cutting G.R., Sharma N. (2022). Genetics of Cystic Fibrosis: Clinical Implications. Clin. Chest Med..

